# Laparoscopic transperitoneal repair of retrocaval (circumcaval) ureter

**DOI:** 10.1590/S1677-5538.IBJU.2017.0409

**Published:** 2018

**Authors:** Kaan Gokcen, Gokce Dundar, Gokhan Gokce, Emin Yener Gultekin

**Affiliations:** 1 Cumhuriyet University Faculty of Medine, Department of Urology, Sivas, Turkey; 2 Cizre State Hospital, Department of Urology, Cizre, Turkey

## INTRODUCTION

Retrocaval ureter occurs because of the persistence of the posterior cardinal veins during embryologic development; as an anomaly of inferior vena cava. This can cause varying degrees of ureteral obstruction and surgical intervention is often necessary. We herein report a case of laparoscopic transperitoneal repair of retrocaval ureter.

## PATIENT AND METHODS

A 36-year-old female patient presented with recurrent attacks of flank pain of two years duration. Ultrasound showed right hydronephrosis and dilatation of proximal ureter. Intravenous urography showed right grade 2 hydronephrosis, and the “reverse J” shape of the collecting system suggested retrocaval course of the ureter. Magnetic resonance urography showed grade 2 obstruction due to hooking of the proximal ureter around the inferior vena cava. Laparoscopic transperitoneal retrocaval ureter repairment was planned for our patient. Patient was placed under general anesthesia. A Foley catheter No. 16 Fr size was inserted. After pneumoperitoneum was established in right flank position, three 10 mm trocars were placed including one camera port. Retroperitoneal region visualised through the incision of toldt line and medialisation of the ascending colon. 5 mm trocar was placed for convenience to retraction and dissection. After exposing the retroperitoneum, the ureter was identified coursing posterior to the inferior vena cava. The dilated ureter was identified and dissected out up to the lateral border of IVC. The lower ureter also was mobilized in the inter-aortocaval region. The retrocaval portion was also dissected out and mobilized. The proximal ureter was divided at the ureteropelvic junction and transposed anteriorly. The proximal ureter was spatulated and prepared for ureteropelvic anastomosis. A stay suture was taken from the proximal ureter, which stabilized the ureter. The posterior side of ureteropelvic anastomosis performed with a 4.0 vicryl suture in a continuous fashion. The anastomosis was continued on the anterior side with a new suture. A 4.8 French 26 cm double j stent was placed through the 14 French Amplatz dilator before completing the anastomosis. Amplatz dilator was passed through the right 10mm trocar so that the distal portion of the double j stent passed therethrough could be more easily manipulated within the surgical field. We have reduced the stent placement time to a minimum with this necessary method, which was not previously described in the literature. A closed suction drain was placed on the site and the operative site was not retroperitonealized. The surgery was uneventful, with no operative complications or evidence of intra-abdominal bleeding.

According to the publications in the literature, it is noted that some of the urologists place a double J stent before the procedure. Preoperative ureteral stenting may facilitate ureteric identification but we believe that the double j stent inside an ureter may limit the mobilization of an ureter, especially the retrocaval portion.

## RESULTS

The duration of the surgery was 75 minutes. The amount of bleeding was 28mL. On the postoperative 2nd day, the urethral catheter was removed and the patient was discharged on the third day postoperatively. Stent removal was done on the 3rd postoperative week and retrograde pyelogram showed normal ureter. Post-operative follow up with ultrasound showed that hydronephrosis had regressed.

## CONCLUSIONS

Laparoscopic transperitoneal repair of retrocaval ureter is useful and feasible, with minimal invasiveness and an early post-operative recovery. Laparoscopic transperitoneal procedure may be preferable for retrocaval ureters.

## ARTICLE INFO

**Available at: http://www.intbrazjurol.com.br/video-section/20170409_Gokcen_et_al**

**Int Braz J Urol. 2018; 44 (Video #10): 649-50**

